# Convergence of cortical and thalamic input to direct and indirect pathway medium spiny neurons in the striatum

**DOI:** 10.1007/s00429-013-0601-z

**Published:** 2013-07-06

**Authors:** Icnelia Huerta-Ocampo, Juan Mena-Segovia, J. Paul Bolam

**Affiliations:** Medical Research Council Anatomical Neuropharmacology Unit, Department of Pharmacology, University of Oxford, Mansfield Road, Oxford, OX1 3TH UK

**Keywords:** Basal ganglia, Corticostriatal, Thalamostriatal, Synapses, Neostriatum

## Abstract

The major afferent innervation of the basal ganglia is derived from the cortex and the thalamus. These excitatory inputs mainly target the striatum where they innervate the principal type of striatal neuron, the medium-sized spiny neurons (MSNs), and are critical in the expression of basal ganglia function. The aim of this work was to test directly whether corticostriatal and thalamostriatal terminals make convergent synaptic contact with individual direct and indirect pathway MSNs. Individual MSNs were recorded in vivo and labelled by the juxtacellular method in the striatum of BAC transgenic mice in which green fluorescent protein reports the expression of dopamine D1 or D2 receptors. After recovery of the neurons, the tissue was immunolabelled for vesicular glutamate transporters type 1 and 2, as markers of cortical and thalamic terminals, respectively. Three of each class of MSNs were reconstructed in 3D and second-order dendrites selected for electron microscopic analysis. Our findings show that direct and indirect pathway MSNs, located in the matrix compartment of the striatum, receive convergent input from cortex and thalamus preferentially on their spines. There were no differences in the pattern of innervation of direct and indirect pathway MSNs, but the cortical input is more prominent in both and synaptic density is greater for direct pathway neurons. The 3D reconstructions revealed no morphological differences between direct and indirect MSNs. Overall, our findings demonstrate that direct and indirect pathway MSNs located in the matrix receive convergent cortical and thalamic input and suggest that both cortical and thalamic inputs are involved in the activation of MSNs.

## Introduction

The major afferent innervation of the basal ganglia is derived from the cortex and the thalamus and is carried principally by the glutamatergic corticostriatal and the thalamostriatal pathways. Virtually, the whole of the cortical mantle projects onto the striatum in a highly topographically organised manner providing motor and cognitive information, and indeed imparting functionality onto the striatum. The corticostriatal system has been the subject of intense investigation and is often considered as the principal ‘driver’ of the striatum and the basal ganglia in general. The thalamostriatal system, which mainly originates in the intralaminar nuclei and provides the basal ganglia with ascending sensory, attentional and salience information, is also critical in the expression of basal ganglia function. Although identified in the early studies of basal ganglia connectivity (Kemp and Powell 1971), the thalamostriatal system is often not considered in studies of basal ganglia organisation or function. Several lines of evidence indicate that the thalamostriatal system is likely to be as functionally important as the corticostriatal system in the expression of basal ganglia function.

On the basis of immunostaining for molecular markers of the corticostriatal and thalamostriatal pathways, i.e. vesicular glutamate transporters types 1 and 2 (VGluT1 and VGluT2, respectively), it has been shown that the numbers of synapses in the striatum arising from the two regions are of the same order of magnitude although cortical input is more abundant than thalamic input (Lacey et al. 2005; Raju et al. 2006). In a similar manner to the corticostriatal projection, thalamostriatal neurons target striatal medium spiny projection neurons (MSNs) (Dube et al. 1988; Xu et al. 1991; Sadikot et al. 1992; Lacey et al. 2007; Doig et al. 2010; Lei et al. 2013) and several types of striatal interneurons (Lapper and Bolam 1992; Rudkin and Sadikot 1999; Sidibe and Smith 1999) and electrophysiological analyses reveal that striatal MSNs are sensitive to thalamic stimulation (Kocsis et al. 1977; Vandermaelen and Kitai 1980; Ding et al. 2008; Smeal et al. 2008; Nanda et al. 2009; Sciamanna et al. 2012; Ellender et al. 2013). Both corticostriatal and thalamostriatal synapses are in a position to be similarly modified by the dopaminergic nigrostriatal pathway (Moss and Bolam 2008) and are differentially modulated by histamine (Ellender et al. 2011).

A fundamental principle of the organisation of the basal ganglia is that MSNs are divided into two main types on the basis of their targets and expression of various molecular markers. The so-called ‘direct pathway’ MSNs directly innervate the output nuclei of the basal ganglia [internal segment of the globus pallidus (GPi) and substantia nigra pars reticulata (SNr)], whereas ‘indirect pathway’ MSNs take an indirect route to the output nuclei that includes the external segment of the globus pallidus (GPe) and the subthalamic nucleus (STN). Using BAC transgenic mice in which eGFP is expressed under the D1 or D2 dopamine receptor promoters (to label direct and indirect pathway MSNs, respectively) (Gong et al. 2003; Valjent et al. 2009), we recently demonstrated that corticostriatal and thalamostriatal terminals make synaptic contact with direct and indirect pathway MSNs to a similar degree and provided indirect evidence of convergence of cortical and thalamic terminals on individual MSNs (Doig et al. 2010), observations that are consistent with electrophysiological analyses (Kocsis et al. 1977; Vandermaelen and Kitai 1980; Ding et al. 2008; Smeal et al. 2008; Nanda et al. 2009; Sciamanna et al. 2012; Ellender et al. 2013).

In view of the importance of the two excitatory drives to the striatum and the fundamental separation of direct and indirect pathway MSNs, we sought to test directly whether corticostriatal and thalamostriatal terminals make convergent synaptic contact with individual direct and indirect pathway MSNs. Our approach was to perform in vivo juxtacellular labelling of MSNs in the D1 and D2 BAC transgenic mice together with immunolabelling for VGluT1 and VGluT2 and quantitative electron microscopic analyses.

## Materials and methods

All experimental procedures were performed on male BAC transgenic mice in which the presence of EGFP reports the expression of either dopamine D1 or D2 receptors (Mutant Mouse Regional Resource Centres, MMRRC; hereafter referred to as D1 and D2 mice) and were conducted in accordance with the Animals (Scientific Procedures) Act, 1986 (UK) and Oxford University Ethical Review Process.

### Electrophysiological recordings and data analysis

D1 and D2 EGFP mice (30–40 g) were anaesthetised with urethane (1.6 g/kg, i.p. ethyl carbamate, Sigma) and anaesthesia was maintained with supplemental doses of urethane (0.16 g/kg). All wound margins were infiltrated with the local anaesthetic, bupivacaine (0.75 % w/v; Astra, Kings Langley, UK), and corneal dehydration was prevented by the application of Hypromellose eye drops (Norton Pharmaceuticals). Mice were placed in a Harvard apparatus mouse stereotaxic frame, and body temperature was maintained at 37–38 °C using a homeothermic heating device and a rectal thermometer. Anaesthesia levels were assessed from the electrocorticogram (ECoG) and by testing reflexes to a cutaneous pinch of the hind paw. The ECoG was recorded via a 0.3 mm diameter steel screw juxtaposed to the dura mater above the frontal cortex (3.25 mm anterior and 2.0 mm lateral to bregma; Franklin and Paxinos 2008) and was referenced against an electrode placed above the cerebellum. The raw ECoG signal was band-pass filtered (0.3–1,500 Hz, −3 dB limits) and amplified (2,000×; DPA-2FS filter/amplifier; Scientifica, Harpenden, UK) before acquisition. Extracellular recordings of action potentials of individual striatal neurons were made using 15–25 MΩ glass electrodes (tip diameter ~1.5 μm), filled with saline solution (0.5 M NaCl) and neurobiotin (1.5 % w/v, Vector Laboratories Ltd., Peterborough, UK). Electrode signals were amplified (10×) through the active bridge circuitry of an Axoprobe-1A amplifier (Molecular Devices Corp., Sunnyvale, CA, USA), AC-coupled and amplified a further 100× (NL-106 AC–DC Amp: Digitimer Ltd., Welwyn Garden City, UK), before being band-pass filtered between 0.3 and 5 kHz (NL125: Digitimer). A Humbug (Quest Scientific) was used to eliminate mains noise at 50 Hz. All biopotentials were digitised online with a PC running Spike2 acquisition and analysis software (version 5; Cambridge Electronic Design, Cambridge, UK). Striatal neuronal activity was recorded, first, during cortical slow-wave activity (SWA) which accompanies deep anaesthesia and is similar to the activity observed during natural sleep, and during episodes of cortical activation which contain patterns of activity that are more analogous to those observed during the awake, behaving state (Steriade 2000).

Electrophysiological data from 11 neurons (five D1-MSNs and six D2-MSNs) during coincident cortical SWA were analysed offline. The waveforms of the extracellular action potentials were measured from the beginning of the positive deflection to the lowest point of the negative trough to quantify their biphasic duration. Action potentials were then digitised and converted to a time series of events using in-built Spike2 functions. The spontaneous discharge of striatal neurons was measured during epochs of robust SWA to determine the mean firing rate. To identify the preferred phase of discharge of striatal neurons during cortical slow oscillations, the ECoG signal was low-pass filtered (<5 Hz) using a Butterworth filter and applied a Hilbert transform to obtain the instantaneous phase angle (Saleem et al. 2010). The mean phase angle and vector length were obtained using the Matlab Circular Statistics Toolbox (Berens 2009).

### Juxtacellular labelling of single neurons

To identify and locate the recorded neurons and enable an analysis of their morphological properties, they were labelled with neurobiotin by the juxtacellular method following the electrophysiological recordings (Pinault 1996; Bevan et al. 1998). A microiontophoretic current was applied (1–10 nA positive current, 200 ms duration, 50 % duty cycle) when the electrode was juxtaposed to the recorded neuron, as evaluated by the amplitude of the action potential (from 1 to 4 mV, typically 2 mV). Reliable labelling was obtained when the firing of the neuron was robustly modulated by the current injection for 1–5 min. The neurobiotin was left to be transported along neuronal processes for 2–6 h. They were then given a lethal dose of urethane and intracardially perfused with ~20 mL of 0.01 M PBS at pH 7.4, followed by 60 mL of 3 % w/v paraformaldehyde and 0.1 % w/v glutaraldehyde in 0.1 M PB at pH 7.4. Brains were post-fixed in the same fixative for 2–3 h and stored in PBS at 4 °C until sectioned.

### Fluorescent imaging of recorded neurons

Brains were sectioned at 50 μm in the parasagittal plane using a vibrating microtome (VT000S; Leica Microsystems). To identify and visualise the labelled neurons, the neurobiotin was revealed by incubation with Cy3-conjugated streptavidin (1:1,000 in PBS, 4 °C overnight; Zymed). Sections containing the somata, and dendritic and axonal profiles, were incubated in a cryoprotectant solution (0.05 M phosphate buffer, 25 % sucrose, 10 % glycerol) overnight, then freeze-thawed twice in liquid nitrogen to increase penetration of the reagents. The sections were washed thoroughly and then incubated in 10 % normal goat serum (NGS; Vector Laboratories) in PBS for 2 h at room temperature. Sections containing the somata were used to confirm the nature of the neurons using a chicken anti-GFP antibody (1:500, Aves Labs), followed by incubation in goat anti-chicken secondary antibody conjugated to Alexa 488 (1:500, Invitrogen). They were then incubated in a rabbit anti-μ opioid receptor antibody (1:1,000 in PBS-NGS 1 % at 4 °C overnight; Immunostar) followed by incubation in donkey anti-rabbit secondary antibody conjugated to Cy3 (1:400, Jackson) to determine the location of the neurons with respect to the patch/striosome (μ-opioid receptor-rich) and matrix (μ-opioid receptor-poor) compartments of the striatum. Recorded and labelled neurons that were immunopositive for GFP were selected for further study. However, in one case, we included a GFP-immunonegative MSN from a D2 mouse that had axonal projections to SNR and was thus categorised as a direct pathway MSN.

### Permanent peroxidase labelling and immunolabelling for VGluTs

The neurobiotin-filled neurons were then revealed using permanent peroxidase reactions by incubating all sections in an avidin–biotin–peroxidase complex (ABC Elite; Vector Laboratories) for 3–4 h at room temperature. The sections were then washed in PBS followed by washes in Tris-buffer (0.5 M, pH 8; TB). Diaminobenzidine (DAB, 0.025 % and 0.5 % nickel ammonium sulphate in TB) was added to the sections and incubated for 15 min. The peroxidase reaction was initiated by the addition of H_2_O_2_ to a final concentration of 0.01 %. The reaction was allowed to continue for 5–7 min and was stopped by several washes in TB and then PB. Alternate sections were then incubated in a rabbit antibody against VGluT1 (1:1,000, MAb Technologies, Stone Mountain, GA) or either a rabbit or a guinea pig (somata section) antibody against VGluT2 (1:2,000, Synaptic Systems, Göettingen, Germany) in PBS-NGS 1 % overnight at room temperature. Sections incubated in the rabbit antibodies against VGluT1 and VGluT2 were then incubated with a goat antibody against rabbit IgG (1:100 in PBS-NGS 1 %; Dako, High Wycombe, UK) overnight at 4 °C, followed by a 3–4 h incubation in rabbit peroxidase–antiperoxidase (PAP) (1:100 in PBS-BSA; Dako) at room temperature. Sections incubated in the guinea pig VGluT2 antibody were incubated with a secondary biotinylated goat antibody against guinea pig IgG (1:500, Vector Labs) for 3–4 h at room temperature, followed by incubation in avidin–biotin–peroxidase complex (ABC Elite; Vector Laboratories) for 3–4 h at room temperature. In both cases, the bound peroxidase was then revealed by incubation in 0.025 % DAB with 0.006 % H_2_O_2_ as substrate.

The VGluT primary antibodies were raised against rat VGluT1 and VGluT2 (amino acids 543–560 and 510–582, respectively). The distribution of immunolabelling at the light and electron microscopic level was distinct for each primary antibody and consistent with previous observations by ourselves (Lacey et al. 2005; Moss and Bolam 2008; Doig et al. 2010) and others (Fremeau et al. 2001; Herzog et al. 2001; Kaneko and Fujiyama 2002; Fujiyama et al. 2004, 2006; Raju et al. 2006, 2008), using the same or different antibodies. No immunolabelling was observed following omission of the VGluT1 or VGluT2 antibodies or when tissue from wild-type littermates (D1/D2 BAC-EGFP negative) was processed using the anti-GFP antibody.

### Preparation of tissue for electron microscopy

The sections were then placed flat in glass Petri dishes and post-fixed in osmium tetroxide (1 % in PB; Oxkem, Oxford, UK) for 15–20 min. They were washed in PB and dehydrated through a graded series of dilutions of ethanol with 1 % uranyl acetate (TAAB, Reading, UK) included in the 70 % ethanol solution to increase contrast in the electron microscope. Following absolute ethanol, sections were washed twice in propylene oxide (Sigma) for 15 min and placed into a resin overnight at room temperature (Durcupan ACM, Fluka, Gillingham, UK). They were then mounted in resin on glass microscope slides and polymerized at 60 °C for 48h.

### Light microscope analysis and 3D reconstructions

All sections were examined in the light microscope to determine the extent of dendritic and axonal labelling. The peroxidase reaction for neurobiotin gave rise to a blue–black reaction product, whereas the VGluT1- and VGluT2-immunoreactive structures were identified by a dense brown precipitate in small puncta that were distributed throughout the whole striatum. At high magnification it was possible to identify close appositions between VGluT1- and VGluT2-immunoreactive boutons and dendritic spines of the individually labelled MSNs (Fig. 2). Light microscopic images at different magnifications were taken as an aid to light and electron microscopic correlation and as a guide to select the areas for re-embedding.

All six neurons included in this study were completely and homogeneously filled with the reaction product without fading at dendritic ends. Local axon collaterals and long collaterals leaving the striatum were observed for each reconstructed neuron. The whole dendritic arbour of all six neurons was fully reconstructed in three dimensions (100× oil immersion objective) using Neurolucida (MicroBrightField), a Nikon Eclipse microscope equipped with an x, y, z motorised stage and Lucivid (MicroBrightField). The axons leaving the striatum were reconstructed for one MSN from a D1 mouse and one from a D2 mouse. As labelled dendrites were densely surrounded by VGluT-positive punctate structures it was difficult to clearly identify spines unless a clear point of origin on the dendritic shaft was observed. For this reason, we did no attempt to quantify spines.

The spliced final 3D reconstruction was corrected for shrinkage in each dimension: x (6.3 %), y (6.0 %) and z (8 %) (Sadek et al. 2007). Quantitative data relating to dendritic parameters for each reconstructed neuron were obtained using the Neurolucida Explorer software. Scholl analyses were performed with radius segments of 20 μm. All the data were exported to Excel and submitted to statistical analysis. In addition to this, the numbers of primary dendrites arising from the two populations of MSNs were quantified in fluorescently labelled sections of ten additional neurons.

Once the neuron was fully reconstructed and the dendritic order for each dendritic fragment within the sections determined, second-order dendrites present at the top or bottom of the section from tissue processed for VGluT1 and VGluT2 were selected. They were cut from the slides and re-embedded in a cylinder of resin for subsequent ultrathin sectioning for the electron microscope.

### Electron microscope analysis

Serial sections, (~55 nm) were cut on an ultramicrotome (Leica EM UC6, Leica Microsystems) and collected on pioloform-coated, single-slot copper grids (Agar Scientific, Stansted, UK). The sections were lead-stained for 5 min and examined in a Philips CM100 electron microscope. The dendrites of the labelled neurons were identified in the electron microscope in the first section on a grid and examined in each consecutive serial section. Each of the labelled dendritic profiles in each of the serial sections was digitally recorded at an indicated magnification of 5.2× and 8,900× using a Gatan multiscan CCD camera. Presynaptic terminals forming synapses with the spines or dendritic shafts of the labelled MSN were identified in the images and categorised as VGluT-immunopositive or -negative over the serial sections. This process was carried out until ten synapses were identified in tissue labelled for VGluT1 and VGluT2 for each dendrite. The postsynaptic targets were characterised, but due to the dense peroxidase reaction product in the postsynaptic structure it was not possible to make unequivocal distinctions between asymmetric (Gray’s type 1) and symmetric (Gray’s type 2) synapses although it is well established that VGluT1- and VGluT2-positive terminals form asymmetric synapses in the striatum (Fujiyama et al. 2004, 2006; Lacey et al. 2005; Raju et al. 2006, 2008 Moss and Bolam 2008; Doig et al. 2010). The digital images were analysed and measurements made using the image-processing package, Fiji (win 32-bit), and they were adjusted for contrast and brightness using Adobe Photoshop CS3. No adjustments were made for shrinkage of the tissue as a consequence of electron microscope sectioning.

### Statistical analysis

Data were assessed for normality using the Shapiro Wilson test. All statistical calculations and graphs were made using the software SigmaPlot (version 12.0). For all the anatomical data, a two-way ANOVA with repeated measures was performed and Tukey post hoc comparisons were used to determine differences within and between direct and indirect pathway neurons. In addition, a contingency table was made and data submitted to a *z* test to analyse the proportions of VGluT1 and VGluT2 innervation onto direct and indirect MSNs. The estimation of total number of synapses between direct and indirect pathway MSNs was compared using a *t* test. The electrophysiological data was analysed using non-parametric tests (Mann–Whitney *U*). Differences were considered significant when *P* < 0.05.

## Results

### In vivo firing properties of direct and indirect pathway MSNs

**Fig. 1:**
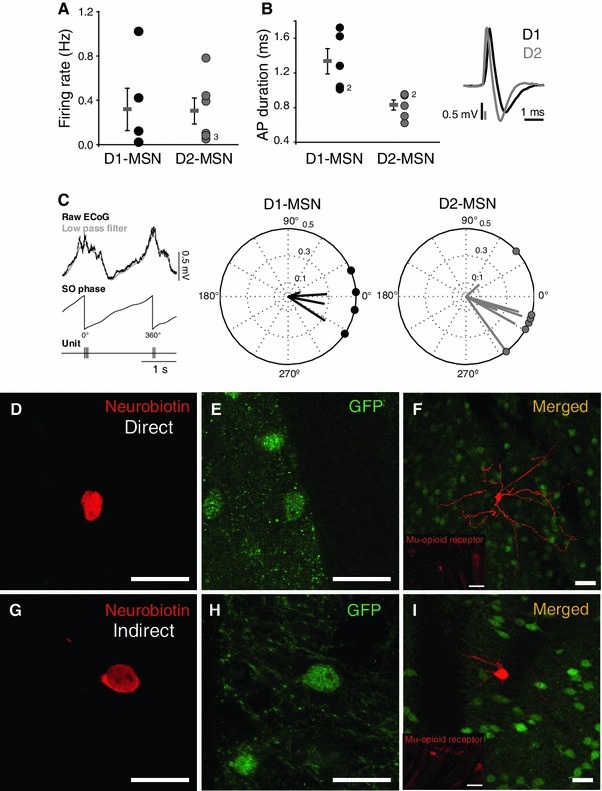
Morphological and physiological properties of identified direct and indirect pathway MSNs recorded in vivo. **a** The firing rates of direct (D1) and indirect pathway (D2) MSNs during cortical SWA were not significantly different. **b** Action potential duration was significantly shorter in D2-MSNs than in D1-MSNs (*P *< 0.05; *inset*: trace of the average waveform of representative examples of each group). **c** Slow oscillations were filtered and the instantaneous phase obtained to calculate the preferred phase of the slow oscillation in which the MSNs fired. Both D1- and D2-MSNs tend to fire around 0°, which corresponds to the UP state of the slow oscillations. **d–i** The neurons were filled with neurobiotin (*red*, **d**, **g**) and tested for immunoreactivity for GFP (*green*, **e**, **h**) revealing them as direct and indirect pathway neurons in the D1 and D2 BAC transgenic mice, respectively. The merged images (**f**, **i**) show the same neurons at lower magnification. Immunolabelling for μ-opioid receptors (*red* insets in **f**, **i**) confirmed the location of the recorded neurons in the matrix compartment (μ-opioid receptor-poor regions). *Scale bars* **d–i** 20 μm,* insets* in **f** and **i** 100 μm

Extracellular recordings of the spontaneous activity of D1- and D2-MSNs were made in a total of 11 neurons that were subsequently labelled by the juxtacellular method. All neurons in the electrophysiological analysis were confirmed to be MSNs by the high density of spines in their dendritic arbours. Putative MSNs were initially recognised by their low basal firing rates of 0.1–1 Hz during SWA. We observed a wide variability in the firing rate of MSNs during stable SWA. Both D1- and D2-MSNs tended to fire one or more (bursting) spikes per slow oscillation cycle for a few cycles, and then remain silent for variable periods of time. No differences in the firing rate between groups were detected (Fig. 1a), but the duration of the action potentials was shorter in D2-MSNs (*P* < 0.05; Fig. 1b). Even though the activity of MSNs was highly irregular, it tended to organise during the cortical UP states (i.e. when the ECoG signal showed its highest amplitude during SWA). Thus, we observed that both D1- and D2-MSNs fire during the same phase of the cortical slow oscillations (Fig. 1c), although the vector size for D2-MSNs tended to be larger than for D1-MSNs (i.e. D2-MSNs appear to be more strongly coupled to the slow oscillations). During periods of cortical activation (absence of slow oscillations), MSNs lost their periodicity and changed their firing pattern (data not shown).

### Light microscopic observations

**Fig. 2:**
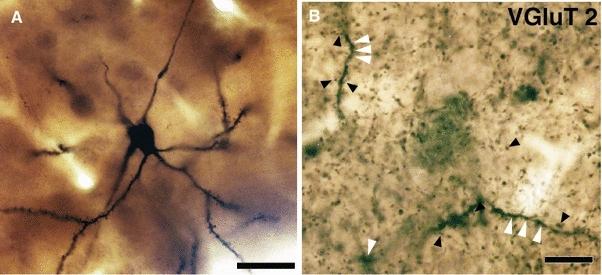
**a** Light microscopy of the peroxidase staining of an indirect pathway MSN. **b** The surface of the section showing dendrites of the neuron (some indicated by white *arrowheads*) within the dense field of VGluT2-positive varicosities derived from the thalamus (some indicated by *black arrowheads*). *Scale bars* 20 μm

**Fig. 3:**
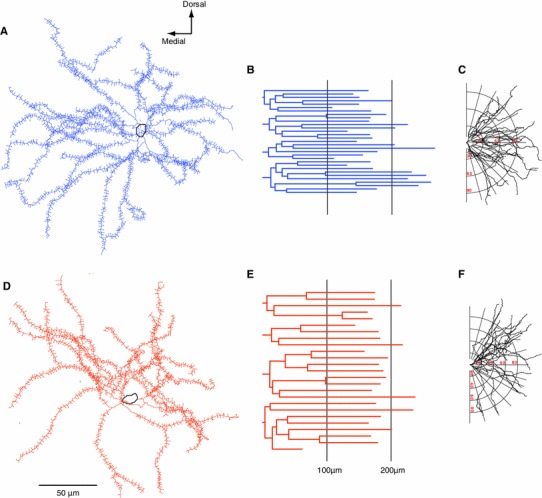
Three-dimensional reconstructions and dendrograms of representative direct and indirect pathway MSNs. The somata, dendrites and spines (approximate location of spines indicted) of identified direct (**a**) and indirect (**d**) pathway MSNs were fully reconstructed. Their corresponding dendrograms (**b** and **c**, **e** and **f**, respectively) display the length, orientation, number and branching points of each dendrite of the same neurons. Note that both neurons have the same number of primary dendrites; however the direct pathway MSN, in this case, possesses more dendritic fragments (**b**, 58) than the indirect pathway MSN (**e**, 46). The fan dendrograms (torsion ratio of 1.19) show that there are no differences in the dendritic orientation between direct (**c**) and indirect (**f**) pathway MSNs

All neurons that were recorded from, labelled with neurobiotin and subsequently localised in the sections (Fig. 1d, e, g, h) were located in the matrix compartment of the striatum as defined by the low expression of μ-opioid receptor immunoreactivity (inset in Fig. 1f, i). Neurons that possessed the electrophysiological characteristics of MSNs showed the typical features of MSNs in the fluorescently labelled and peroxidase-labelled sections. Thus, they possessed a medium-sized cell body that gave rise to dendrites that were initially spine-free and then became densely laden with spines (Figs. 1–3). There was no apparent difference in the morphological features of direct and indirect pathway MSNs. VGluT1- and VGluT2-immunolabelling was localised to small punctate structures (Fig. 2), the distribution of which in the cortex and basal ganglia was consistent with previous studies (for references see above). Close appositions between VGluT1 and VGluT2 immunopositive boutons and the high or low endings (i.e. in the region of penetration of the immunoreagents) of direct and indirect pathway MSN dendrites were frequently observed.

Three neurons of the two subtypes of MSN, identified by the expression of immunoreactivity for EGFP or in one case by the absence of labelling, and located in the dorsolateral striatum, were selected on the basis of the labelling of their dendritic tree, spines and axons for further detailed analysis.

### Morphological characteristics of direct and indirect pathway MSNs

**Fig. 4:**
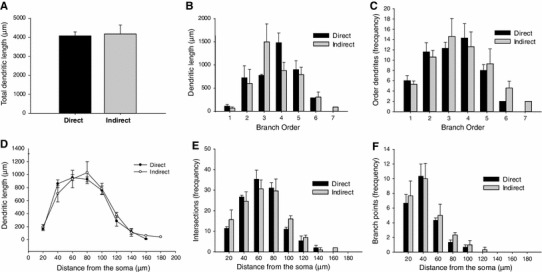
Morphological analysis of the dendrites of reconstructed direct and indirect pathway MSNs. The total dendritic length is similar between direct and indirect pathway MSNs (**a**). On average, direct and indirect MSNs display comparable patterns of dendritic branching order (**c**) with similar length (**b**, **d**). The Scholl analysis of the reconstructed dendrites shows that there are no differences in the average length within each concentric 20-μm radial sphere from the somata (**d**), the number of intersections (**e**) and the number of branch points (**f**) between direct and indirect MSNs

The 3D reconstructions of the somata and dendrites of the recorded neurons show that, on average, direct and indirect MSNs possess similar dendritic architectures (Fig. 3). Overall, no differences were observed between direct and indirect MSNs in the number of primary dendrites (D1 MSN 5.3 ± 0.66; D2 MSN 5 ± 0.57; *P* = 0.61, mean ± SEM, *n* = 8 neurons per group; ANOVA two-way repeated measures in this and subsequent comparisons; data from both fluorescently labelled and peroxidase-labelled sections), nor the number of higher-order dendrites (Fig. 4c). Similarly, there was no difference between direct and indirect pathway MSNs in the average length of dendrites of different orders (Fig. 4b), the total dendritic lengths (D1 4084.26 ± 204 μm, D2 4176 ± 471.2 μm; *P* = 0.87) (Fig. 4a) and the preferred orientation of their dendritic trees (Fig. 3c, f). Furthermore, we did not observe differences in dendritic length at increasing distances from the soma (Fig. 4d), the number of intersections, which neither correlates with the overall density of the dendritic arbour nor the total number of branch points (Fig. 4e, f) between the two types of MSNs.

### Electron microscopic observations

#### Convergence of cortical and thalamic terminals onto direct and indirect pathway MSNs

**Fig. 5:**
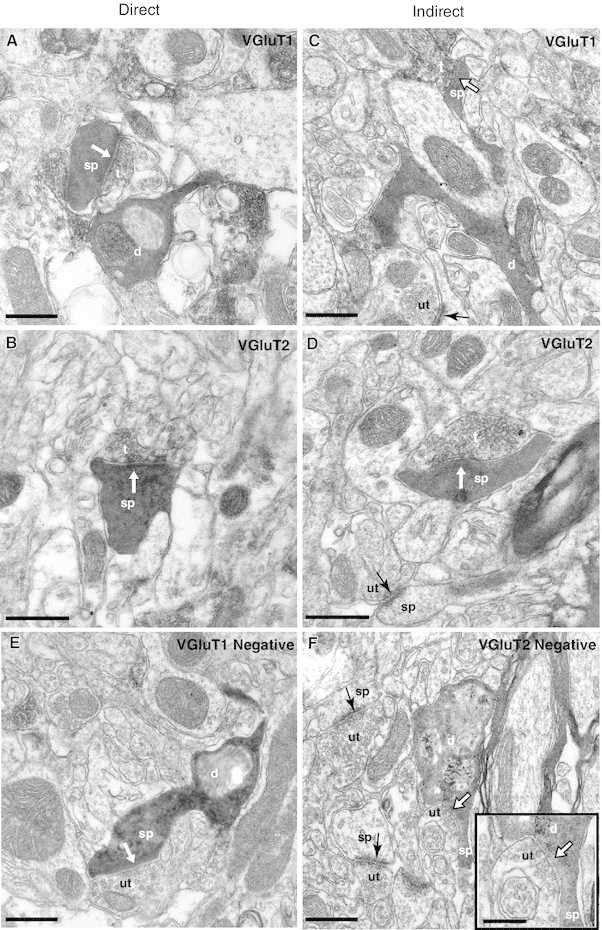
Individual direct and indirect pathway MSNs receive convergent VGluT1-positive (cortical) and VGluT2-positive (thalamic) synaptic input. Dendrites (d) and spines (sp) of an individually labelled direct pathway MSN (**a**, **b**, **e**) and an individually labelled indirect pathway (**c**, **d**, **f**). MSNs were identified by the dense black reaction product in the cytoplasm. **a**, **b** Spines (sp) of the direct pathway MSNs receive synaptic input (*white arrows*) from both VGluT1-positive **(a)** and VGluT2-positive **(b)** terminals (t). (**e**) A spine of the same neuron receives input (*white arrow*) from a VGluT1-negative terminal (ut) which is likely to be of thalamic origin. **c**, **d** Spines (sp) of the indirect pathway MSN also receive synaptic input (*white arrows*) from both VGluT1-positive (**c**) and VGluT2-positive (**d**) terminals (t). (**f**) A spine of the same neuron receives input (*white arrow*) from a VGluT2-negative terminal (ut) which is likely to be of cortical origin. Note also the unlabelled terminals (ut) forming synapses (*black arrows*) with unlabelled spines in (**B**) and (**F**). *Scale bars* 200 nm,* inset* in F 100 nm

**Fig. 6:**
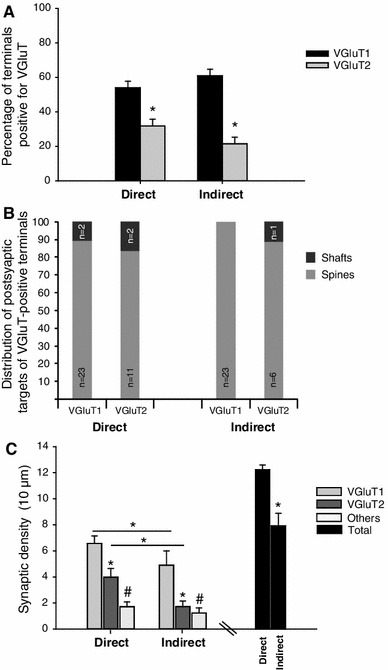
Quantitative electron microscope analysis of the cortical and thalamic innervation of individual direct and indirect MSNs. **a** Histograms show the average percentage (±SEM) of afferent synaptic terminals of second-order dendrites of neurobiotin-labelled MSNs that were VGluT1- and VGluT2-positive. On average, both, direct and indirect MSN dendrites receive significantly more cortical than thalamic synaptic input (two-way ANOVA with repeated measures; *P = 0.003). **b** VGluT1- and VGluT2-positive terminals preferentially target dendritic spines of both subtypes of MSNs. **c** The overall density of afferent synapses (*t* test *P = 0.014), and the density of both cortical and thalamic afferent synapses, was greater on direct pathway MSNs than indirect pathway MSNs (two-way ANOVA with repeated measures; *P = 0.014). Within the direct and indirect pathway MSN populations there is a greater cortical innervation when compared to thalamic and innervation from terminals negative for both of the VGluTs (two-way ANOVA with repeated measures;^ #^ *P* = 0.001)

Electron microscopic analysis revealed that the juxtacellularly labelled MSNs visualised with Ni-DAB, were densely filled with an electron-dense reaction product that adhered to the internal surface of the plasmalemma and sometimes filled subcellular organelles (Fig. 5). VGluT1- and VGluT2-immunoreactive terminals, visualised with DAB, were identified by the presence of a lighter reaction product and synaptic vesicles (Fig. 5a–d). The labelled dendrites were examined in each of the serial sections and afferent synapses were noted and characterised. For the calculation of synaptic densities, the length of dendrite analysed was calculated from the 3D light microscopic reconstructions. A mean of 69 sections were analysed for each dendrite which, on average, was equivalent to ~14 μm. For each dendritic fragment, the distance to the soma was estimated to further classify them as proximal or distal dendrites. All dendrites included for EM analysis were defined as distal dendrites as they were located within a distance >20 % of the longest possible distance from the soma to any dendritic ending. In agreement with previous studies (Fujiyama et al. 2004, 2006; Lacey et al. 2005; Raju et al. 2006, 2008; Moss and Bolam 2008; Doig et al. 2010; Lei et al. 2013), the majority of VGluT1- and VGluT2-positive terminals made synaptic contact with the spines, as opposed to dendritic shafts, of MSNs (92 and 100 % of VGluT1-positive synaptic boutons in contact with spines of direct and indirect MSNs, respectively, 84.6 and 85.7 % VGluT2-positive boutons in contact spines of direct and indirect MSNs, respectively) (Fig. 6b).

We found that the dendrites of individual direct (Fig. 5a, b) and indirect pathway MSNs (Fig. 5c, d) receive convergent synaptic input from both VGluT1-positive (cortical) and VGluT2-positive (thalamic) presynaptic terminals. Of a total of 47 synapses identified in contact with dendrites of direct pathway MSNs in VGluT1-immunolabelled sections, 25 were established by VGluT1-immunopositive terminals (Figs. 5a, 6a). In sections labelled for VGluT2, dendrites of the same neurons were observed to form 40 synapses, and 13 of the presynaptic terminals were immunopositive for VGluT2 (Figs. 5b, 6a). The number of VGluT1-positive synaptic terminals (as a proportion of total synapses; 53.9 ± 2.1 %; *n* = 3) in contact with direct pathway MSNs was significantly greater than the proportion positive for VGluT2 (31.8 ± 3 %; *n* = 3; two-way ANOVA, 1 factor repetition, *P* < 0.003). Similarly, for indirect MSNs, the proportion of synaptic terminals that were VGluT1-positive (23 out of 38 synaptic boutons, 60.8 ± 5.8 %, *n* = 3) (Figs. 5c, 6a) was significantly greater than the proportion that was VGluT2-positive (7 out of 34 synaptic boutons, 21.4 ± 3.5 %, *n* = 3, two-way ANOVA, 1 factor repetition, *P* < 0.003) (Figs. 5d, 6a).

Overall, these findings indicate that second-order dendrites of both direct and indirect pathway MSNs receive convergent synaptic input from both the cortex and the thalamus. In both cases, the number of synaptic terminals from the cortex was significantly greater than the number of synaptic terminals from the thalamus. However, there were no significant differences between direct and indirect MSNs in their innervation by VGluT-positive terminals (two-way ANOVA, 1 factor repetition, *P* = 0.13).

The overall average synaptic density on second-order dendrites was slightly higher for direct MSNs (12.26 ± 0.36 synapses per 10 μm) than for indirect MSNs (7.95 ± 0.96 synapses per 10 μm; *t* test, *P* = 0.01) (Fig. 6c). The average density of VGluT1-positive synaptic terminals was 6.57 ± 0.58 and 4.97 ± 1.1 synapses per 10 μm for direct and indirect pathway MSNs, respectively. VGluT2-positive synaptic terminal density was 3.98 ± 0.67 and 1.73 ± 0.42 synapses per 10 μm for direct and indirect pathway MSNs, respectively (Fig. 6c). The analysis revealed that direct pathway MSNs receive a greater density of both cortical and thalamic synapses than indirect pathway MSNs (two-way ANOVA, 1 factor repetition; *P* = 0.014). Nevertheless, within the indirect pathway MSNs group, there is a greater number of VGluT1-positive synapses than VGluT2-positive synapses per 10 μm of dendritic fragment analysed (Fig. 6c). The density of unlabelled synaptic terminals was similar between MSNs subtypes (1.71 ± 0.37 and 1.23 ± 0.39 synapses per 10 μm for direct and indirect pathway MSNs, respectively).

There were no differences in the average cross-sectional area of VGluT1 and VGluT2 presynaptic terminals that innervate direct (VGluT1, 0.20 ± 0.05 μm^2^; VGluT2, 0.15 ± 0.01 μm^2^) and indirect (VGluT1, 0.19 ± 0.04 μm^2^; VGluT2, 0.27 ± 0.08 μm^2^) pathway MSNs. The length of the synaptic membrane was similar for direct (VGluT1, 0.19 ± 0.03 μm; VGluT2, 0.18 ± 0.04 μm) and indirect (VGluT1, 0.26 ± 0.03 μm; VGluT2, 0.29 ± 0.03 μm) MSNs. Furthermore, there were no differences in the average diameter of corticostriatal terminals contacting both subtypes of MSNs (direct, 0.83 ± 0.12 μm, *n* = 25; indirect, 0.844 ± 0.11 μm, *n* = 23). However, the diameter of VGluT2-positive terminals establishing synapses with indirect pathway MSNs (0.5 ± 0.03 μm, *n* = 7) was twofold greater than those contacting direct pathway MSNs (1 ± 0.09 μm, *n* = 13) implying that their shapes are different.

## Discussion

The main findings of the present study are firstly, MSNs located in the matrix compartment of the striatum that give rise to the direct and indirect pathways of information flow through the basal ganglia, receive synaptic input from cortical and thalamic terminals. Secondly, individual direct and indirect pathway MSNs receive convergent synaptic input from both the cortex and thalamus. Thirdly, MSNs of both pathways receive a significantly larger number of terminals from the cortex than the thalamus although they were of a similar order of magnitude. Finally, 14–17 % of terminals were negative for both of the VGluTs (which include those forming both symmetrical and asymmetrical synapses) and are likely to consist of GABAergic terminals derived from other MSNs, GABA interneurons and the GPe as well as dopaminergic terminals derived from the substantia nigra pars compacta, cholinergic terminals derived from local interneurons and glutamatergic terminals that do not express VGluT1 or 2. Our findings thus extend previous anatomical studies (Xu et al. 1991; Lacey et al. 2005; Raju et al. 2006; Doig et al. 2010; Lei et al. 2013) that provided indirect evidence of convergence and electrophysiological studies that demonstrated responsiveness of individual MSNs to cortical and thalamic stimulation (Kocsis et al. 1977; Vandermaelen and Kitai 1980; Ding et al. 2008; Smeal et al. 2008; Nanda et al. 2009; Sciamanna et al. 2012; Ellender et al. 2013). We conclude that there is no significant difference in the pattern of innervation of direct and indirect pathway MSNs and that it is likely that activation of direct and indirect pathway MSNs is a consequence of the timed and patterned firing of afferent synapses from both the cortex and thalamus that provide different functional information, together with the patterned activity of other afferent synapses. Thus, one of the central roles of the striatum, and in particular of MSNs, is to integrate the large number of glutamatergic cortical and thalamic afferents, provide synaptic processing and filtering and ultimately control the output of the striatum, which in turn controls the activity of other basal ganglia structures and ultimately influences behaviour.

### Technical considerations

The use of the BAC transgenic mice in which EGFP is driven by D1R or D2R promoters has proven to be a pivotal tool in the study of the striatum in relation to the direct/indirect pathway model of organisation and flow of information through the basal ganglia (DeLong 1990). These mouse lines have been used extensively, primarily in in vitro studies, to elucidate the properties of the two subpopulations of MSNs (Gong et al. 2003; Kreitzer and Malenka 2007; Surmeier et al. 2007; Cepeda et al. 2008; Gertler et al. 2008; Bertran-Gonzalez et al. 2010; Doig et al. 2010; Ellender et al. 2011). The second innovation that enabled us to address the issues of convergence was the demonstration that subtypes of the VGluTs are selective markers of cortical and thalamic synaptic terminals. Thus in the striatum, VGluT1 is selectively expressed by corticostriatal terminals, whereas VGluT2 is selectively expressed by thalamostriatal terminals (Fremeau et al. 2001; Herzog et al. 2001; Kaneko and Fujiyama 2002; Fujiyama et al. 2004; Raju et al. 2006, 2008; Barroso-Chinea et al. 2008). Although there is a small population of thalamic neurons that express low levels of VGluT1, these are unlikely to contaminate the present and previous findings as the low level of expression is not likely to be detected at the level of the striatum (Barroso-Chinea et al. 2007, 2008). These molecular tools together with the ability to record and label individual MSNs in their entirety in vivo and examine the same neurons in the electron microscope enabled us to address directly the innervation of direct and indirect pathway MSNs by the cortex and thalamus. Using this approach our results confirmed and extended previous findings, demonstrating that corticostriatal and thalamostriatal terminals innervate both, direct and indirect pathway MSNs (Doig et al. 2010; Lei et al. 2013) and we also demonstrated directly, that individual direct or indirect pathway MSNs receive input from both the cortex and the thalamus.

A caveat of our analysis, however, was that because of the labour-intensive nature of the electron microscopy we confined our study to second-order dendrites only. We chose second-order dendrites because this is the point at which the dendrites of MSNs become densely laden with spines and because of the relative ease of identification in correlated light and electron microscopic studies. It remains to be established whether the rules of innervation of higher-order dendrites are similar.

The analysis of individual neurons at the electron microscopic level also allowed us to address the issue of the homogeneity of the glutamatergic innervation of the striatum. Our data demonstrates that MSNs receive a higher proportion of their glutamatergic innervation from cortical as opposed to thalamic terminals (see also Lei et al. 2013). However, there was no difference between the pattern of innervation of the two subtypes of MSNs. It is interesting to note that, when expressed as a density of synapses, we observed a preferential thalamic and cortical innervation of direct pathway MSNs over indirect pathway MSNs. This tendency has been suggested previously in monkeys (Sidibe and Smith 1996) and in rats (Lei et al. 2013) and raises the possibility that direct pathway MSNs are under a greater thalamic influence that indirect pathway MSNs.

### Striatal heterogeneity

Functional heterogeneity is imposed on the striatum by the topography of the corticostriatal, thalamostriatal and other pathways, and the striatum is highly heterogeneous with respect to the variety of interneuron subtypes in addition to the subtypes of MSNs (Kawaguchi et al. 1990; Kawaguchi 1993; Fujiyama et al. 2011; Sharott et al. 2012) that have unique roles in the microcircuitry. These levels of heterogeneity are overlain by another level of heterogeneity in which molecular markers, and both inputs and outputs are organised in a compartmental fashion referred to as the striosome/patch and matrix compartmentalization (see Gerfen and Bolam 2010). Indeed, the input from the parafascicular nucleus of the thalamus was one of the first projections to be recognised to respect to the striosome/patch-matrix boundaries (Herkenham and Pert 1981) and it is clear that the synaptic organisation of the thalamostriatal projections are ordered by this level of heterogeneity (Fujiyama et al. 2006; Raju et al. 2006). In order to control for this, we restricted ourselves to the analysis of neurons within the matrix compartment to reduce the number of variables and because of the difficulty in labelling neurons within the striosome.

### Heterogeneity of thalamostriatal projections

Although there are many similarities between the cortical and thalamic innervation of the striatum in terms of overall synaptic organisation, the innervation of MSNs and relationship to dopamine terminals (Lacey et al. 2005; Raju et al. 2006; Doig et al. 2010; Moss and Bolam 2008), it is clear that the thalamostriatal projection is highly heterogeneous. Besides arising from different parts of the thalamus and giving rise to different synaptic organisation within the striatum (Smith et al. 2004), combined electrophysiological and anatomical analyses in rat (Lacey et al. 2007) have demonstrated that the properties of thalamostriatal neurons in the rostral intralaminar thalamus (central lateral nucleus; CL) are markedly different from those in the caudal intralaminar thalamus (parafascicular; Pf). Parafascicular neurons possess reticular-like dendritic arbours, discharge groups of spikes at relatively low frequencies and some preferentially innervate striatal dendrites and others preferentially innervate spines. In contrast, CL neurons have bushy dendrites, exhibit low-threshold calcium spike bursts and preferentially establish synapses with dendritic spines. Furthermore, striatal synapses arising from these two populations of thalamic neurons have different functional properties (Ellender et al. 2013). In the present study, we used a molecular marker of the population of thalamostriatal synapses (i.e. VGluT2), so we are not able to determine the relationship of subtypes of thalamostriatal neurons to individual MSNs. We do know from tracing studies that input from the Pf preferentially innervates dendritic shafts, a large proportion of which are interneurons (Lapper and Bolam 1992; Rudkin and Sadikot 1999); however, further work will be necessary to elucidate the cell-specific innervation of identified neurons in the striatum.

### Axonal and dendritic architecture of direct and indirect pathway MSNs

The labelling of individual MSNs in vivo and the post hoc immunochemical characterisation and reconstructions enabled us to address issues relating to the axonal and dendritic architecture of direct and indirect pathway MSNs. Single axon reconstructions in rats and monkeys have shown that although there is a clear separation of axon targets of direct and indirect pathway MSNs, direct pathway MSNs also give rise to minor collaterals that innervate the GPe (Kawaguchi et al. 1990; Parent et al. 1995; Wu et al. 2000; Fujiyama et al. 2011). We show here that this is also the case for mouse MSNs; we noted a GFP-immunonegative MSN in the D2 mouse (i.e. a direct pathway MSN) with projections to the SNr and minor collaterals to the GPe. The axons of the remaining MSNs included in this study were not reconstructed; however, we recorded axon projections in the GPe and output nuclei of the basal ganglia. In the case of the D2 EGFP immunopositive MSNs (*n* = 3), we observed that the axon ended and branched in GPe, whereas projections of D1 EGFP immunopositive MSNs (*n* = 2) were observed in the SNR but also both gave few collaterals to the GPe.

We observed no difference in the number of primary dendrites between MSN subpopulations nor did we observe differences in the mean number and length of dendrites as function of branch order and the total surface area and volume. These findings are not consistent with the observation of Gertler et al (2008) who observed the difference in the morphological properties of direct and indirect pathway MSNs that may underlie differences in electrophysiological properties. The reasons for these differences remain to be established but may relate to the small number of observations we made (except for numbers of primary dendrites), the age of the animals and the fact that our analyses were confined to the matrix.

## Conclusions

Our findings demonstrate directly that individual direct and indirect pathway MSNs in the mouse striatum receive convergent synaptic input from both the cortex and the thalamus. We also show that MSNs of both pathways receive a greater input from cortex than thalamus. We thus suggest that the selection of the ensemble of MSNs that fire during a basal ganglia-associated behaviour is a consequence of activity in corticostriatal neurons carrying motor and cognitive information and activity in thalamostriatal afferents carrying information on saliency, wakefulness, etc. Perhaps also, the selection of motor programmes, as is proposed for the direct pathway, requires more ‘thalamic’ information than does the suppression of motor behaviours, as is proposed for the indirect pathway. Of course it remains to be established whether the relative proportions of cortical and thalamic innervation could vary throughout the dendritic domains of an individual MSNs as our analyses were confined to second-order dendrites.
